# Quantifying the invasion risk of West Nile virus: Insights from a multi-vector and multi-host SEIR model

**DOI:** 10.1016/j.onehlt.2023.100638

**Published:** 2023-10-08

**Authors:** Martina Ferraguti, Afonso Dimas Martins, Yael Artzy-Randrup

**Affiliations:** a Departamento de Biología de la Conservación y Cambio Global, Estación Biológica de Doñana (EBD), CSIC, C/Américo Vespucio, 26, E-41092 Seville, Spain; b Department of Theoretical and Computational Ecology (TCE), Institute for Biodiversity and Ecosystem Dynamics (IBED), University of Amsterdam, Science Park 904, 1098 XH Amsterdam, the Netherlands; c Department of Population Health Sciences, Faculty of Veterinary Medicine, University of Utrecht, Yalelaan 7, 3584 CL Utrecht, the Netherlands; d CIBER of Epidemiology and Public Health (CIBERESP), Madrid, Spain

**Keywords:** Epidemiology, Feeding preferences, Mathematical modelling, Mosquito community, *Passer domesticus*, Vector-borne diseases

## Abstract

The invasion of vector-borne diseases depends on the type of specific features of the vector and hosts at play. Within the *Culex pipiens* complex, differences in ecology, biology, and vector competence can influence the risk of West Nile virus (WNV) outbreaks. To determine which life-history traits affect WNV invasion into susceptible communities the most, we constructed an epidemiological Susceptible-Exposed-Infectious-Recovered model with three vector (eco)types, *Culex pipiens pipiens, Cx. pip. molestus*, and their hybrids, and two vertebrate hosts, birds (as amplifying hosts) and humans (as dead-end hosts). We investigated how differences in feeding preferences and transmission rates influenced WNV transmission across different habitats and two seasons (Spring versus Summer), to investigate the impact of increasing mosquitoes on the WNV transmission risk. Our results showed that vector feeding preferences and the transmission rate between mosquitoes and birds were the parameters that most influenced WNV invasion risk. Even though our model did not predict WNV invasion across any of the studied environments, we found that natural habitats displayed the highest susceptibility to WNV invasion. *Pipiens* (eco)type acted as the primary vector in all habitats. Hybrids, contrary to common opinion, showed minimal involvement in WNV transmission. However, it is important to interpret our study results with caution due to the possibility of idealized spring and summer seasons being reflected in the field-collected data. Our study could be a tool to enhance current vector surveillance and control programs by targeting specific vector types in specific environments, especially in natural habitat, which are most responsive to environmental shifts. The joint approach based on epidemiological modelling based on field collected data can help to reduce wasted time and economic costs while maximizing the efficiency of local public health authorities.

## Highlights

• Natural habitat showed the highest WNV invasion risk.
• *Culex pipiens* (eco)type dominates WNV amplification.
• Vector feeding preferences and transmission rate of infection between mosquitoes and birds contribute the most to changes in *R*_*0*_ estimates.
• Hybrids poorly contribute to WNV invasion into susceptible communities.

## 1 Introduction

The number of vector-borne diseases threatening humans is currently on the rise [1]. The risk of pathogen transmission dynamics usually depends on the interactions between the insect vector, the disease agent and the vertebrate host, being the system influenced by the environmental and ecological drivers where these interactions take place [2]. An increase in the number of competent vector types (i.e., able to transmit a disease [3]), each with a unique identity and requirements determined by its ecology, habitat eligibility and feeding preference (ornithophilic vs. mammophilic), can expand the range of infected hosts.

Nowadays, modelling attempts explicitly considering the identity of vector species identity are relatively rare. We investigated the sensitivity of different environments and seasons, to vector control of the West Nile virus (WNV), one of the most widespread emerging zoonotic arboviruses distributed worldwide [4] and a major public health concern due to its unprecedented human outbreaks during the last decade [5]. WNV is a multi-vector / multi-host pathogen maintained in a mosquito-bird transmission cycle, where *Culex pipiens* mosquitoes - a polytypic species native to Europe, belonging to a species complex (or assemblage) [6] - are the primary vectors in the United States and Europe [7], and humans and other mammals are incidental “dead-end” hosts [8].

*Culex pipiens* is considered to be a plastic species with two morphologically identical forms or (eco)types, named *Cx. pipiens pipiens* [Linnaeus, 1758] (hereafter *pipiens*) and *Cx. pipiens molestus* [Forskål, 1775] (hereafter *molestus*). While both (eco)types display similar vector competence for pathogen transmission (see [9] for a recent review on the status of *Cx. pipiens* complex), they have been found to exhibit distinctly different ecological and behavioural characteristics (Table 1). However, it is known that *pipiens* and *molestus* can hybridise [[9], [10], [11]] and produce fertile offspring in captivity, begging the question of how they remain distinct in nature [12]. Besides, the difference in their ecological requirements and feeding behaviour are key features in our study due to the vector implications [10,13].

**Table 1:** Examples of ecological and biological differences between the two *Cx. pipiens* (eco)types.

Ecological and biological characteristics	*pipiens* (eco)type	*molestus* (eco)type
Habitat suitability	Aboveground natural habitats	Man-made belowground habitats
Behaviour	Diapause in winter	Active year-round
Feeding preference	Bird-biting	Human-biting
Copulating behaviour	Mating in swarm, open spaces	Mating in confined spaces (stenogamy)
Need blood for oviposition	Blood meal required to develop eggs	First clutch can be laid before blood meal (autogeny)

From an epidemiological perspective, *pipiens* play a significant role in the transmission of WNV between birds and humans in natural habitat. Conversely, *molestus* are considered more influential in urban areas. Hybrid mosquitoes, presumably characterised by an intermediate host preference and habitat requirements, are commonly assumed to be the most high-risk vectors for bridging the transmission of WNV from birds to humans [14]. The hybridization between *pipiens* and *molestus* has been identified as a major factor influencing WNV transmission [15]. The patchy distribution of the *Cx. pipiens* complex across the Mediterranean basin, with variations in ecology and behaviour in different environments, may explain the irregularity of WNV outbreaks in Europe. The mixed feeding behaviour resulting from hybridization increases the risk of mosquito populations acting as bridging vectors for WNV transmission [16]. However, the specific roles of the three *Culex* (eco)type mosquitoes in WNV transmission, as well as the impact of *pipiens-molestus* hybridization on pathogen transmission to humans, still require further investigation.

Using a mathematical model, we investigated the transmission implications of three different types of vectors, namely *pipiens* mosquitoes, *molestus* mosquitoes, and their hybrids, along with two vertebrate hosts, birds as competent hosts and humans as dead-end hosts. The mosquitoes compete within and between types and differ in feeding preferences. Our study aimed to explore how parameters associated with i) type-related vector feeding patterns, ii) vector and host life-history traits (e.g., birth rates and mean longevity), and iii) vector-pathogen, host-pathogen, or vector-host-pathogen- interactions (e.g., transmission rate and the mean duration of infection), could potentially affect WNV epidemiology across a natural-rural-urban gradient.

Currently, the role of variations among vector species, particularly in the context of mosquito-borne diseases, has received limited attention in disease epidemiological models, despite its importance in the medical and ecological fields [17] (see Supplementary Material A for the State-of-the-art). In this study, we have developed a Susceptible-Exposed-Infectious-Recovered (SEIR) model for WNV transmission, considering a system with multiple vector and multiple host species based on field-collected data collected in southern Spain. Our aim is to address three key questions:

1) Are there specific environments, such as urban, rural and natural habitats, where targeting a particular vector species is crucial for effective WNV control?
2) Are there any seasonal effects related to mosquito abundance that influence WNV transmission in these environments?
3) What is the quantitative impact of hybrid mosquitoes on the propagation of WNV?

To answer these questions, we examine the invasion risk of WNV by assessing the basic reproduction number, *R*_*0*_, which represents the expected number of secondary cases generated by a single infectious individual in a susceptible population [18]. Since estimates of *R*_*0*_ are influenced by mosquito specific parameters and their relative distribution in different habitats, we employ a mathematical model to quantify the respective contributions of each vector and host type-related parameter to the invasion risk of WNV.

## 2 Materials and methods

### 2.1 A general multi-vector and multi-host model

**Fig. 1:**
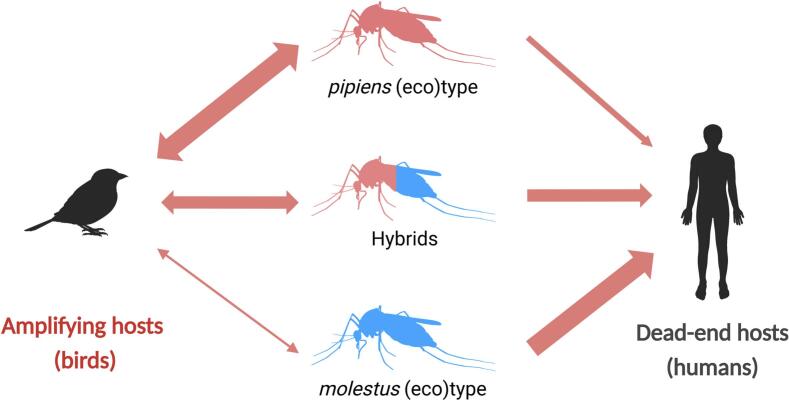
Schematic representation of the enzootic transmission pathways of WNV. The persistence of WNV depends on the co-occurrence of both competent vectors and amplifier hosts (birds), which can get infected and transmit the virus onwards, while humans are an incidental host, as they can get infected and suffer clinical disease, but are not able to replicate the virus and hence transmit it onwards (i.e., dead-end hosts). As such, humans do not contribute to maintaining an ongoing cycle of transmission. The thickness of the arrows indicates the relative feeding preference of the three different mosquito types.

We developed a multi-vector and multi-host model that extends the SEIR epidemiological framework [19]. This model allows for a more realistic representation of WNV transmission pathways by incorporating multiple types of vectors and hosts. In our model, we consider three distinct types of vectors, each with different feeding preferences: ornithophilic mosquitoes (which preferentially feed on birds, such as the *pipiens*), mammophilic mosquitoes (which preferentially feed on mammals, such as the *molestus*), and generalist feeders (mosquitoes with no specific preference, such as hybrid descendants). Furthermore, we include two types of hosts: competent hosts, which can become infected and transmit the virus to susceptible mosquitoes (birds), and incompetent hosts, which can become infected but are unable to transmit the pathogen (humans) (Fig. 1). For a more detailed description of the models, as well as the parameter values used for the derivation of the *R*_*0*_, please refer to Table 2, Supplementary Material B, and Fig. S1.

**Table 2:** Parameters used in the numerical examples and respective sources. Values are taken for *pipiens*, *molestus*, and their hybrid mosquitoes as the vectors, and birds (*Passer domesticus*) and humans as the hosts.

Parameter	Description	Estimate	Reference
*ν*_*p*_	Birth rate of *pipiens*	0.537	[64]
*ν*_*m*_	Birth rate of *molestus*	0.537	[64]
*ν*_*h*_	Birth rate of hybrids	0.537	[64]
*ν*_*b*_	Birth rate of birds	0.023	[65]
*ν*_*d*_	Birth rate of humans	5.5 × 10^−5^	[66]
*μ*_*p*_	Death rate of *pipiens*	0.029	[64]
μ_m_	Death rate of *molestus*	0.029	[64]
*μ*_*h*_	Death rate of hybrids	0.029	[64]
*μ*_*b*_	Death rate of birds	0.0015	[67]
*μ*_*d*_	Death rate of humans	3.91 × 10^−5^	[68]
*c*_*ii*_	Effect of competition within (eco)type *i*	0.02	⁎
*c*_*ij*_	Effect of competition from subtype *j* on (eco)type *i*	0.01	⁎
*d*_*ii*_	Fraction of competition from (eco)type *i* that affects the death rate of (eco)type of *i*	0.02	⁎
*d*_*ij*_	Fraction of competition from (eco)type *j* that affects the death rate of (eco)type of *i*	0.01	⁎
*β*_*pb*_	Transmission rate from infected *pipiens* to birds	0.8	[22]
*β*_*mb*_	Transmission rate from infected *molestus* to birds	0.8	[22]
*β*_*hb*_	Transmission rate from infected hybrids to birds	0.8	[22]
*β*_*pd*_	Transmission rate from infected *pipiens* to humans	0.88	[65]
*β*_*md*_	Transmission rate from infected *molestus* to humans	0.88	[65]
*β*_*hd*_	Transmission rate from infected hybrids to humans	0.88	[65]
*β*_*bp*_	Transmission rate from infected birds to *pipiens*	0.16	[22]
*β*_*bm*_	Transmission rate from infected birds to *molestus*	0.12	[22]
*β*_*bh*_	Transmission rate from infected birds to hybrids	0.09	[22]
*σ*_*p*_	Transition rate from exposed to infected *pipiens*	0.106	[20]
*σ*_*m*_	Transition rate from exposed to infected *molestus*	0.106	[20]
*σ*_*h*_	Transition rate from exposed to infected hybrids	0.106	[20]
*σ*_*b*_	Transition rate from exposed to infected birds	0.5	[23]
*σ*_*d*_	Transition rate from exposed to infected humans	0.25	[23]
*α*_*b*_	WNV-induced additional death rate of birds	0.1	[70]
*α*_*d*_	WNV-induced additional death rate of humans	5 × 10^−7^	[69]
*γ*_*b*_	Recovery rate in birds	0.18	[20]
*γ*_*d*_	Recovery rate in humans	0.91	[71]

⁎ Assumed values.

We assume that the transmission of the virus occurs solely between vectors and hosts, neglecting horizontal transmission and vertical transmission events, as these are sporadic in the case of WNV [20]. Therefore, all newly added individuals through birth are considered susceptible. WNV transmission is assumed to be frequency-dependent, meaning that the number of blood meals taken by a mosquito per unit of time is limited and independent of host population size [21]. We assume an equal biting rate for the three vector types [22], but they differ in their feeding preferences. Constant birth and mortality rates are also considered for both hosts and vectors. In addition to the natural mortality rate, vectors also experience the effects of competition, both within the same vector type and between different vector types. Similarly, hosts also suffer from disease-induced mortality.

### 2.2 The case-study

The inclusion of three vector and two host types in our framework allows for the analysis of more non-linear dynamics in WNV transmission (see Supplementary Material C for further details on model parametrization). This approach allows us to investigate the following aspects:

1) Assessing the impact of varying feeding preferences among vector types on the risk of WNV invasion in different habitat types. By considering the unequal feeding preferences of the vector types, we can assess how this variability influences the spread of the virus.
2) Evaluating the relative contribution of each vector type to the risk of WNV invasion within each habitat. This assessment was based on their corresponding *R*_*0*_ values. These analyses provide insights into the role of specific vector and host life-history traits in the transmission of the virus within a given habitat.
3) Identifying the most significant factors in the multi-vector and multi-host system for WNV invasion. By explicitly considering the transmission pathways between multiple vector and host types, we can determine the key drivers of the risk of WNV invasion and assess their relative importance (Fig. S1).

For the development of our mathematical framework, we utilized field data from house sparrows (*Passer domesticus*), which were chosen as competent hosts for the WNV. Sparrows were selected as a model bird species because they serve as reservoirs for WNV [23] and are highly susceptible to mosquito bites [24]. Furthermore, sparrows are widely distributed in urban environments across continents, making them particularly relevant in the context of urban settings [25]. Sparrows play a crucial role in the amplification and transmission of WNV to humans [[26], [27], [28]]. Additionally, we included humans into our model as dead-end hosts to account for their dilution effect, given that they do not develop sufficient viremia to further transmit the pathogen. When mosquitoes bite humans, they essentially ‘waste’ a bite, reducing their potential for onward transmission, thus acting as a deterrent to the further spread of the virus.

### 2.3 Environmental and seasonal characterization

The relative risk of WNV invasion, quantified by *R*_*0*_, was investigated along a natural-rural-urban gradient in southern Spain, a Mediterranean region known for its active WNV circulation [29,30] (see [31] for further details on the environmental characteristics). Three distinct habitat types, as previously described in Martínez-de la Puente et al. [10], were investigated: natural, rural and urban environments. These habitats exhibited variations in the abundances of the three *Cx. pipiens* (eco)types (Table S1), as well as differences in their feeding preference (Table S2). Additionally, differences in the abundances of bird and human populations were also observed (Table S3).

To investigate the temporal aspects of the vector community, we analysed both spring and summer seasons within each of the three habitats, resulting in a total of six study cases. We compared mosquito abundances between the two seasons (for detailed information regarding assumed vector and host abundances see Table S1), with spring data based on vector abundances observed from May 3^rd^ to June 2^nd^, and summer data derived from vector abundances recorded between July 3^rd^ and August 7^th^ [32]. Bird and human abundances, as well as feeding preference, remained consistent across seasons.

### 2.4 Elasticity analysis: Assessing the impact of vector and host parameters on the basic reproduction number

To assess the impact of different vector and host parameters on *R*_*0*_, an elasticity analysis was conducted [33]. The elasticity of *R*_*0*_ with respect to each parameter (*a*) was calculated using Eq. (1):

(1) $$e\left(a\right)=\frac{a}{R_{0}}\sum_{\mathrm{ij}}\frac{\partial R_{0}}{\partial k_{\mathrm{ij}}}\frac{\partial k_{\mathrm{ij}}}{\partial a}$$

Here, *k*_*ij*_ represents the element in row *i* and column *j* of the next-generation matrix. The analysis involved systematically varying each parameter while keeping all other parameters fixed and observing how *R*_*0*_ reacted proportionally to change in parameter *a*.

This analysis was performed for all parameters in each habitat combination, excluding seasonal effects, using the default values shown in Table 2. The resulting values of *e(a)* indicate whether a particular parameter leads to an increase (positive value) or a decrease (negative value) in *R*_*0*_ when varied. All analyses were conducted using the *R* software (version 4.2.0) and the *popbio* package.

### 2.5 Estimation of the mosquito feeding preference

To estimate host feeding preferences, we used the Manly resource selection design index [34], which is based on a ratio that accounts for relative host density as a measure of host availability. This ratio is denoted as the density-based selection ratio *ŵ*_*i*_. The host feeding preference for each vector type was estimated using Eq. (2):

(2) ŵi=oi/π^i

where *o*_*i*_ represents the proportion of utilized host species *i*, and *π̂*_*i*_ represents the proportion of available host species. The determination of mosquito feeding preferences, following the approach of Hamer et al. [35] and Simpson et al. [36], involved estimating *o*_*i*_ as the proportion of blood-fed mosquitoes of each vector type on a specific host (e.g., house sparrows), out of all available host species (see Table S3 for specific parameters). The Manly selection ratio reflects the degree of mosquito feeding preference for a particular host species. A ratio of 1 indicates no preference or random biting, meaning that mosquito feed on host *i* in proportion to its estimated availability. Ratios greater than 1 indicate an overuse of a host species (more frequent feeding than expected by chance), while ratios less than 1 suggest an underuse of a host species (less frequent feeding than expected by chance). In cases where host species were not observed as a source of blood meal sources but were identified in vertebrate surveys, a blood meal value of one was assigned. For host species observed as blood meal sources but not observed in vertebrate surveys, their density was assumed to be equal to the lowest observed host density at each site.

In natural areas where no mosquito bites on sparrows were recorded, default low values for the feeding preference (*ŵ*_*i*_) of the three *Cx. pipiens* (eco)types were assumed based on previous research (Table S2). The absence of recorded bites on house sparrows hindered the precise calculation of feeding preferences. To address the uncertainty associated with mosquito feeding preference estimates, we performed 100 calculations using fixed parameters from Table S3. In each calculation, *ŵ*_*i*_ values were randomly sampled from a normal distribution with means derived from the estimated preferences in Table S2 (*w*_*i*_ ∼ *N*(*ŵ*_*i*_, 0.1)). These calculations accounted for the different host compositions observed in each habitat.

## 3 Results

### 3.1 Relative contribution and seasonal impact of each vector (eco)type on WNV invasion risk in different habitats

**Fig. 2:**
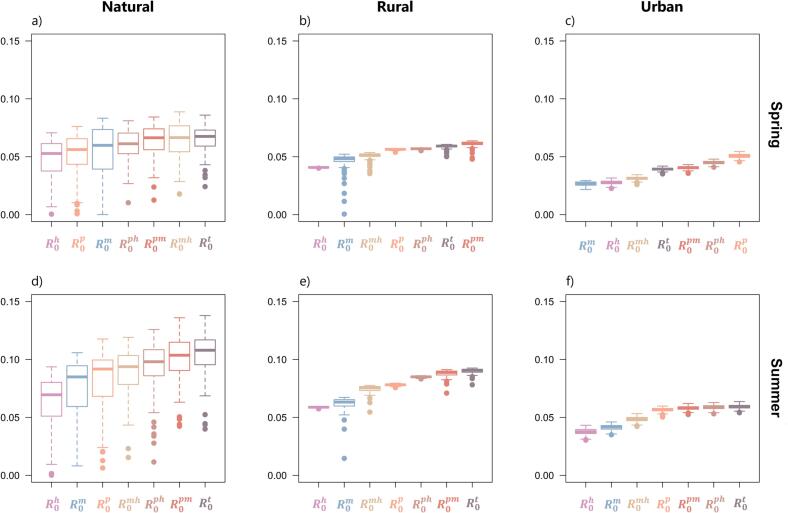
Contributions of each vector (eco)type, and their combinations, to the risk of West Nile virus invasion (expressed by the basic reproduction number *R*_*0*_) in the three habitats: natural (a, d), rural (b, e), and urban (c, f). *R*_*0*_^*h*^ represents the contribution of hybrid mosquitoes alone, *R*_*0*_^*m*^ of *molestus* alone, *R*_*0*_^*p*^ of *pipiens* alone, *R*_*0*_^*mh*^ of *molestus* and hybrids, *R*_*0*_^*ph*^ of *pipiens* and hybrids, *R*_*0*_^*pm*^ of *pipiens* and *molestus*, and *R*_*0*_^*t*^ includes all mosquito (eco)types. Results for Spring (a, b, c), and Summer (d, e, f) seasons, using the species abundances set in Table S1, are shown. Boxplots show the minimum, the interquartile range, the median, and the maximum. Dots represent outliers.

Our findings revealed that WNV had no potential to invade and establish endemicity across all habitats and in both spring and summer seasons, as indicated by *R*_*0*_ values below 1 (Fig. 2). The natural habitat showed the highest invasion risk when all three vector types were present, as indicated by *R*_*0*_^*t*^ values in both seasons (Fig. 2a, d). However, in rural and urban habitats during the Spring, considering all mosquito types resulted in relatively lower *R*_*0*_ values (Fig. 2b, c). Among the vector types, *pipiens* consistently posed the highest risk for WNV invasion, whether analysed alone (*R*_*0*_^*p*^) or in combination with other types (*R*_*0*_^*pm*^*, R*_*0*_^*ph*^).

During the Summer, the susceptibility to WNV invasion noticeably increased in the natural and rural habitats, and to a lesser extent in the urban habitat. However, similar to Spring, WNV did not exhibit the potential to establish endemicity in any of the habitats. In all environments, the highest *R*_*0*_ values were observed when considering all mosquito types (*R*_*0*_^*t*^) (Fig. 2 e, f). This contrasts with the *R*_*0*_ values obtained for the spring season, where the higher mosquito counts resulted in increased competition among vector types, leading to lower transmission.

A method of assessing the contributions of different vector types to human infections is by consulting the elements of the next-generation matrix (see Supplementary Materials B). For example, the value on the first column and fifth row corresponds to the expected number of secondary infections in humans caused by *pipiens*. It was evident that hybrid mosquitoes did not emerge as the primary contributor to the expected number of secondary infections in humans. In natural habitat, infections originating from *pipiens* to humans accounted for 22.4%, while those from *molestus* and hybrids were 40.5% and 37.1%, respectively. Similarly, in rural areas, the proportions were 23.3%, 62.4%, and 14.3% for *pipiens*, *molestus*, and hybrids, respectively. In urban environments, the respective percentages were 44.1%, 20.5%, and 35.4%.

### 3.2 Vector, host and virus specific parameters

**Fig. 3:**
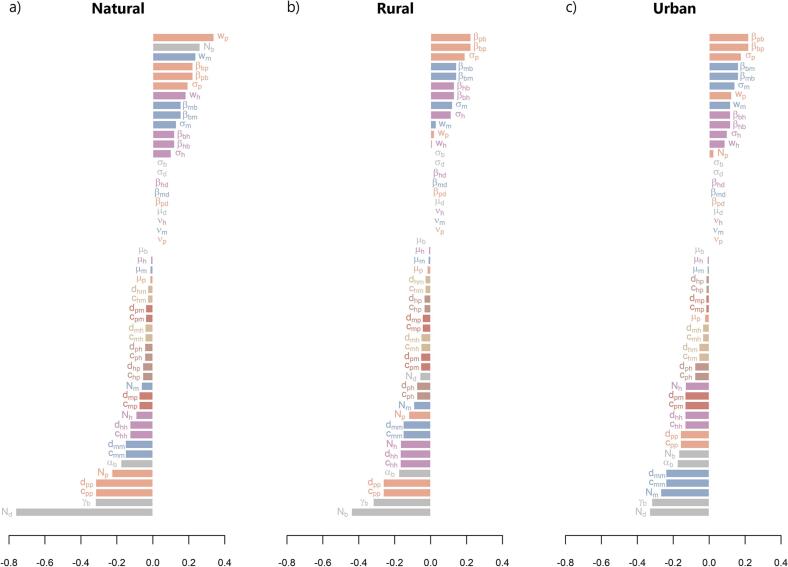
Elasticity of *R*_*0*_ to the lower-level parameters for the three studied habitats, a) natural, b) rural, and c) urban. The default parameters values, for the baseline scenario, as well as their description, are shown in Table 2 and feeding preferences in Table S2.

The elasticity analysis provided information into the relative contributions of different parameters to the risk of WNV invasion (Fig. 3). In the natural habitat, the feeding preference of *pipiens* (*w*_*p*_), which is influenced by local host abundances in the habitat, was found to be the most influential factor facilitating WNV transmission (Fig. 3a). Following that, the availability of avian hosts (*N*_*b*_), the feeding preference of *molestus* (*w*_*m*_), and transmission parameters from *pipiens* mosquitoes to sparrows and back (*β*_*pb*_*, β*_*bp*_), were also important indicators of amplifying the vector-host-pathogen transmission cycle. Conversely, the abundance of dead-end hosts (*N*_*d*_) exhibited the most significant impact, followed by the bird recovery from infection (*γ*_*b*_), in reducing the potential for WNV invasion (Fig. 3a).

In the rural habitat, contrasting with the natural habitat, the parameters associated with mosquito feeding patterns (i.e., *w*_*p*_*, w*_*m*_*, w*_*h*_) of all three vector types showed significantly less influence on the risk of WNV transmission. However, similar to the natural habitat, the parameters related to the *pipiens* remained the most influential in facilitating WNV transmission (Fig. 3b). Overall, virus-related parameters showed a stronger impact on WNV transmission compared to mosquito-related parameters, which played a relatively reduced and more negligible role with minor differences in their contributions. The transmission rate of WNV from *pipiens* to its avian host and back (*β*_*pb*_*, β*_*bp*_), followed by the transition rate from exposed to infected *pipiens* (*σ*_*p*_), and then the transmission rate from *molestus* mosquitoes to birds and back (*β*_*mb*_*, β*_*bm*_), and the transmission from and to the hybrids (*β*_*hb*_*, β*_*bh*_) were the major drivers of virus amplification in the rural environment (Fig. 3b). By contrast, the abundance of birds (*N*_*b*_) and their recovery rate (*γ*_*b*_) had the most significant impact in reducing the risk of WNV invasion. This may initially be a counter-intuitive result, but it is a consequence of the overall vector-to-host ratio leading to higher *R*_*0*_ values rather than the abundance of hosts alone [37]. The patterns observed in the urban environment were similar to those observed in the rural habitat, with the exception that the abundance of dead-end hosts (*N*_*d*_) had the most pronounced impact in reducing the potential for WNV invasion (Fig. 3c).

## 4 Discussion

The impact of vector species on disease risk remains a relatively understudied topic. Traditional mosquito-borne transmission models have often overlooked vector diversity, despite its crucial role in validating assumptions and conclusions of mathematical studies, as pathogen incidence is greatly influenced by vector distribution [17]. In this study, we aimed to assess how different life-history traits associated with three vector types and two vertebrate hosts contribute to the invasion risk of WNV in natural, rural, and urban habitats.

Mosquito species exhibit considerable variations in their ecology, climate preference, and environmental requirements, which are likely to impact their distribution [38] and, consequently, affect the epidemiology of the vector-borne pathogens they transmit [39,40]. Multiple factors can contribute to alterations in vector-host contact ratios, including mosquito feeding preferences and changes in habitat structure [41]. We examined how small differences in vector and host type-specific parameters influenced the likelihood of WNV outbreaks within an ecosystem. Understanding these factors is crucial for predicting and managing the risks of vector-borne diseases.

### 4.1 Impact of habitat characteristics on the basic reproduction number

The impact of land-use change significantly influences the epidemiology of mosquito-borne diseases [42,43]. In our study, we observed the highest risk of WNV invasion in natural habitats (Fig. 2a,d). This is likely attributed to the elevated abundance of amplifying bird species, particularly house sparrows, which serve as preferred hosts for *Cx. pipiens* mosquitoes, thereby facilitating infection and virus transmission. It is worth noting that bird abundance was the second most important parameter influencing the amplification of WNV in natural habitats (Fig. 3a). Conversely, we found that the abundance of dead-end hosts in natural and urban environments (Fig. 3a,c), as well as the abundance of birds in rural habitats (Fig. 3b), along with the duration of infection in birds (*γ*_*j*_), have significant impacts on mitigating the spread of the virus. Specifically, higher populations of humans and sparrows, coupled with faster recovery rates of birds from infection, contribute to limiting WNV transmission acting as a dilution factor by decreasing the overall ratio of vectors to hosts. This effect can be attributed to the modelling of mosquito-bird contacts, and incorporating a time- and space-varying ratio of mosquito-bird contacts into the model may provide more realistic estimates. It is also important to consider the possibility that an excessive number of birds may lead to a scenario where most mosquitoes feed on non-infected hosts [44]. Consequently, these mosquitoes remain uninfected and are unable to contribute to the ongoing virus transmission. Hence, achieving an optimal balance in the abundance of competent hosts is essential for the successful establishment and spread of WNV in rural environment.

These findings highlight the potential effectiveness of managing populations of both competent and non-competent hosts, as well as promoting the prompt recovery of birds from infection, as strategies for reducing the overall spread of the virus and minimizing spill-over to other hosts. By focusing on host population dynamics and recovery rates, interventions aimed at controlling WNV can be tailored to address the specific ecological factors influencing the transmission rate.

Our study also identified vector feeding preference (*w*_*i*_) and transmission rates (*β*_*ij*_) as the key factors influencing WNV transmission across different habitats (Fig. 3). Among the vector types, *pipiens* mosquitoes were the primary contributors to WNV invasion risk in all environments. This is significant because *pipiens*, as the most competent vector for WNV among the three *Cx. pipiens* (eco)types [45], and being ornithophilic species [11], is more likely to become infected and transmit the virus, especially in habitats with abundant bird populations, although they can also feed on other hosts. Among the *pipiens* parameters, feeding preference played the most crucial role in amplifying WNV invasion in natural habitats (Fig. 3a), while transmission rates from *pipiens* to birds predominantly drove amplification in rural and urban environments (Fig. 3b,c).

Urbanization, which is projected to increase the urban population to approximately two-thirds by 2050 [46], has been shown to cause notable changes in the distribution and abundance of mosquito species at the local level [31,47], with implications for disease transmission dynamics [48]. Notably, the increased abundance of *Cx. pipiens* complex mosquitoes in urban areas has been identified as a key factor contributing to the rise in WNV transmission rates [49]. Furthermore, human-induced habitat alterations not only enhance the environmental suitability for *Cx. pipiens* mosquitoes, but also increase the potential for hybridization between different (eco)types of these mosquitoes [50]. In this context, our models provide insights into the role of the three vector types in a changing world and their implications for WNV transmission dynamics. Nevertheless, our calculation did not predict WNV invasion in any habitat. It is important to acknowledge that other ecological factors may come into play, including the presence of additional reservoir hosts or vector species that could potentially support new transmission cycles [51]. These factors can influence virus transmission and sustain WNV circulation even when *R*_*0*_ values for house sparrows alone are below one. It's worth noting that our study primarily focuses on the most common bird species in the study area, specifically house sparrows, which to some extent helps to contextualize our findings [52]. However, this model inherently restricts the generalizability of our findings to other avian hosts.

### 4.2 Impact of season and mosquito community composition on the basic reproduction number

Our findings highlight that considering multiple seasons provides more informative insights compared to relying solely on direct *R*_*0*_ estimates from a single parameterization (Fig. 2). Specifically, during the spring season, *pipiens* mosquitoes acted as the primary WNV vector, either independently in urban habitats or in combination with *molestus* mosquitoes in the rural areas (Fig. 2). In addition to the combination of the three vector types, the presence of *molestus* and hybrid mosquitoes also made a substantial contribution to WNV invasion in natural habitats, thus emphasizing the importance of determining mosquito community composition as a driving factor in the expansion of WNV [17,53,54].

With rising temperatures in Summer, urban species, including those in the *Cx. pipiens* complex, are expected to thrive [22]. These mosquitoes are well-adapted to warmer water temperatures, whether in natural or artificial containers. Therefore, urban areas benefiting from microclimate refugia created by the heat island effect and human-made structures that provide shelter, are expected to foster the proliferation of mosquito species, particularly the *molestus* [9,55]. During the summer season, WNV transmission was primarily driven by the combination of all three *Cx. pipiens* (eco)types across all habitats. Additionally, in urban habitats, the second most influential factor was the combination of *pipiens* with hybrid mosquitoes, while in rural and natural habitats, it was the combination of *pipiens* with *molestus* mosquitoes. This discovery bears significant relevance in southern European regions, such as our study area, where the epidemiology of WNV becomes more complex due to the warmer climatic conditions that facilitate the coexistence of both *pipiens* and *molestus* mosquitoes in aboveground environments [10]. This co-occurrence may potentially lead to increased hybridization [11,56,57], thereby enhancing the dynamics of pathogen transmission [58].

### 4.3 The role of *Culex* hybrid mosquitoes in the WNV invasion

Accurately identifying and distinguishing between the *pipiens* and *molestus* mosquitoes, as well as their hybrids, is crucial for understanding the distribution patterns and transmission potential of WNV. Genetic differences among these vector types can affect their vector competence [58,59], highlighting the need for a comprehensive assessment of their individual transmission potential and blood-feeding behaviour. Hybridization between *pipiens* and *molestus* mosquitoes has been proposed as a mechanism for generating bridge vectors that exhibit intermediate feeding behaviour and potentially drive WNV epidemics in urban areas [15,60,61]. However, the extent of hybridization and its contribution to WNV transmission require quantitative investigation, where mathematical modelling can provide valuable insights for management strategies.

Our study suggests that hybrids have a limited role in increasing the risk of WNV transmission to humans across different habitats. Although WNV transmission often depends on mosquitoes that feed on both birds and mammals, potentially including hybrids, our findings align with previous studies that have provided inconclusive evidence regarding the role of hybrids in pathogen transmission [10]. Further research is needed to better understand the feeding behaviour and transmission potential of hybrid mosquitoes in the context of WNV transmission. Quantifying the degree of hybridization and its impact on feeding preferences and vector competence of *Cx. pipiens* populations would contribute to the development of more accurate models for WNV transmission and improved control strategies.

If hybrids were found to have a significant role in WNV emergence, strategies aimed at urbanization in rural areas could inadvertently increase their presence, potentially posing a public health risk [62]. Nevertheless, our findings indicate that hybrids have minimal direct impact on WNV transmission, but they can enhance the risk of virus invasion when interacting with either *pipiens* or *molestus* mosquitoes in natural or urban environments (Fig. 2). Therefore, it is crucial to consider the interactions between different mosquito types when designing intervention strategies [63]. Additionally, such studies could provide insights into the potential role of other mosquito species as bridging vectors, facilitating the transmission of WNV and other mosquito-borne diseases.

## 5 Conclusions

Our epidemiological model of WNV transmission, incorporating multiple vector and host types, revealed different parameters influencing the risk of invasion based on each mosquito type. Key determinants of WNV transmission across various habitats included mosquito feeding preferences and transmission rates between mosquitoes and birds or from birds to mosquitoes.

During the spring season, we identified *pipiens* mosquitoes as the primary WNV vectors, both independently in urban habitats and in combination with *molestus* mosquitoes in rural areas. Furthermore, we emphasized the substantial impact of mosquito community composition, including the presence of *molestus* and hybrid mosquitoes, on WNV invasion in natural habitats. As we transitioned into the summer season, the influence of rising temperatures became evident, with the combination of all three *Cx. pipiens* (eco)types drove WNV transmission in all habitats.

Notably, our study challenged the common belief that hybrids play a significant role in WNV transmission, as we found minimal involvement in the virus amplification. Nevertheless, it is important to interpret our study findings cautiously, recognizing the potential influence of idealized scenarios present in field-collected data.

In conclusion, we highlight the importance of adopting an integrative approach when investigating WNV invasion under natural conditions. Our methodology combines insights into the life-history traits of vectors and hosts with an understanding of pathogen ecology within and between these communities, while considering how habitat characteristics may influence these interactions.

## Author contributions

M.F. conceived the idea of the study; A.D.M. performed initial calculations, figure preparation, and contributed to the analysis of results. M.F. and A.D.M. wrote the draft of the manuscript. Y.A.R. reviewed part of the draft of the manuscript. All authors read, contributed to, and approved, the final version of the manuscript.

## Declaration of Competing Interest

The authors declare no competing interests.

## Data Availability

Data will be made available on request.

## Appendix A Supplementary data

Supplementary material Image 1
