# Supplementary material for: Quantifying the invasion risk of West Nile virus: Insights from a multi-vector and multi-host SEIR model
Source: One Health. 2023 Oct 8;17:100638. doi: 10.1016/j.onehlt.2023.100638 (PMC10665159; doi:10.1016/j.onehlt.2023.100638)
Supplement: Supplementary file 1 — Supplementary material [file mmc1.pdf]

Appendix A - Supplementary Material

Quantifying the invasion risk of West Nile virus: insights from a multi-vector and multi-host SEIR model

By: Ferraguti M., Dimas Martins A., Artzy-Randrup Y.

A) State-of-the-art 2

B) Model details and derivation of the basic reproduction number (R_0) 2

Figure S1. Flow diagram for the multi-vector and multi-host WNV model 4

Differential equations of the SEIR model 5

C) Model parametrization: incorporating vector and host data for WNV transmission analysis 8

SUPPLEMENTARY TABLES...11

Table S1. *Assumed vector abundance per season and habitat* 11

Table S2. *Mosquito feeding preference indexes* 12

Table S3. *Vector and host species abundances and blood-fed parameters* 13

A) State-of-the-art

Previous studies have mainly focused on a limited number of host species, often overlooking other vertebrate and vector types within the community. However, it is crucial to incorporate vector species into mosquito-borne transmission models to validate assumptions and draw accurate conclusions, as pathogens are largely influenced by vector distribution. Mosquitoes possess the ability to shape both the temporal and spatial patterns of the pathogens they transmit, and the diversity of vector species can significantly impact disease dynamics by influencing pathogen's host range and transmission rates through their feeding preferences. For instance, among the relatively few existing studies that have incorporated vector species within a mathematical framework, it has been suggested that a higher number of vector species within a community can significantly broaden the overall host range of the pathogen. This expansion occurs as additional opportunities for host colonization become available[1]. Our study takes an innovative approach by modelling the impact of three different vector types from the *Cx. pipiens* complex, which serves as the primary vector of West Nile virus (WNV) in the United States and Europe. We incorporate key aspects of mosquito biology and behaviour, particularly emphasizing the importance of considering the feeding preferences of the three vector types. This factor plays a pivotal role in determining the movement of a pathogen within the WNV transmission network, highlighting the intricate relationship between mosquito behaviour and pathogen spread.

B) Model details and derivation of the basic reproduction number (R_0)

The R_0 value was derived from a next-generation matrix[2], which comprehensively represents all possible transmission pathways of WNV, encompassing the various interactions between hosts and vectors. When the calculated R_0 value exceeds 1, it indicates that the virus has the potential to invade a population. Conversely, an R_0 value below 1 suggests that the virus is not expected to successfully establish an invasion. To investigate the relative contributions of different vector types and their combinations to the invasion risk of WNV, we did not consider WNV prevalence at equilibrium[3]. In this study, we assumed that WNV was non-endemic in the population due to the low occurrence of WNV-infected blood-fed mosquitoes, indicating a fully susceptible population.

Our model encompasses four epidemiological states: susceptible (denoted as "S"), exposed (infected but not infectious, denoted as "E"), infectious (denoted as "I"), and recovered

[1] B. Roche, P. Rohani, A.P. Dobson, J.F. Guégan, The impact of community organization on vector-borne pathogens. Am Nat 181 (2013) 1–11.

[2] O. Diekmann, J.A.P. Heesterbeek, M.G. Roberts, The construction of next-generation matrices for compartmental epidemic models. J R Soc Interface 7 (2010) 873–885.

[3] H. Nishiura, B. Hoye, M. Klaassen, S. Bauer, H. Heesterbeek, How to find natural reservoir hosts from endemic prevalence in a multi-host population: A case study of influenza in waterfowl. Epidemics 1 (2009) 118–128.

(immune to re-infection, denoted as "R"). For competent hosts (birds) and the three vector types, we incorporate a latency period for virus incubation (the "E" state). This latency period holds particular significance in the case of mosquitoes, given that the time scales of latency and mosquito lifespan are often similar and may vary with temperature[4]. Hence, some individuals may die after becoming infected but before becoming infectious due to their relatively short lifespan. Dead-end hosts (humans) can only become infected but cannot transmit the infection to others. Therefore, we model them using an SIR (Susceptible-Infectious-Recovered) framework. Hosts are assumed to recover and acquire lifelong immunity after a characteristic duration of infection, although it is acknowledged that immunity may wane in reality. Given the time scale of our analysis, the assumption of lifelong immunity is a reasonable approximation. Since mosquitoes have a relatively short lifespan and remain infected once they contract a disease, our model only includes the SEI epidemiological states by not incorporating a recovery compartment.

We incorporated WNV transmission dynamics between three different vector types: *pipiens* ($N_p = S_p + E_p + I_p$), *molestus* ($N_m = S_m + E_m + I_m$), and their hybrids ($N_h = S_h + E_h + I_h$), as well as two host types: birds as competent ($N_b = S_b + E_b + I_b + R_b$) and humans as dead-end hosts ($N_d = S_d + E_d + I_d + R_d$) (see the full system of equations below, Fig. S1).

The following assumptions were made: every individual (both vectors and hosts) is directly included by birth into its susceptible compartment at a constant rate Δi. Immigration is not considered. All vector species bite at a fixed rate b_i (i.e., number of bites per mosquito in a unit of time). We account for heterogeneous biting patterns using feeding preference coefficients w_i (see equation 1). WNV transmission is frequency-dependent (i.e., the number of contacts is independent of the population size). After infection (either in vectors or hosts), there is an incubation period with an average duration of $1/\sigma_i$ days, after which the infected individual becomes infectious. The fraction of individuals that this period, $\sigma_i /(\sigma_i+\mu_i)$, can transmit the virus to susceptible individuals (host to vector, and vector to host). This incubation period is particularly relevant for modeling vectors, given its significance in their lifespan. We assume that all vector species share an identical latency period ($\sigma_p = \sigma_m = \sigma_h$). We assume that mosquitoes do not recover from the infection, while hosts recover at constant rate γ_i. Recovered individuals remain immune to infection for the rest of their lives. This simplification is reasonable considering the time scales of the study. Vectors are assumed to remain in an infected state until death, with no emigration. Disease-induced mortality by WNV was considered only for hosts (birds and humans), not for vectors (α_i).

---

[4] S.L. Richards, S.L. Anderson, C.C. Lord, C.T. Smartt, W.J. Tabachnick, Relationships between infection, dissemination, and transmission of West Nile virus RNA in *Culex pipiens quinquefasciatus* (Diptera: Culicidae) NIH Public Access. J Med Entomol 49 (2012) 132–142.

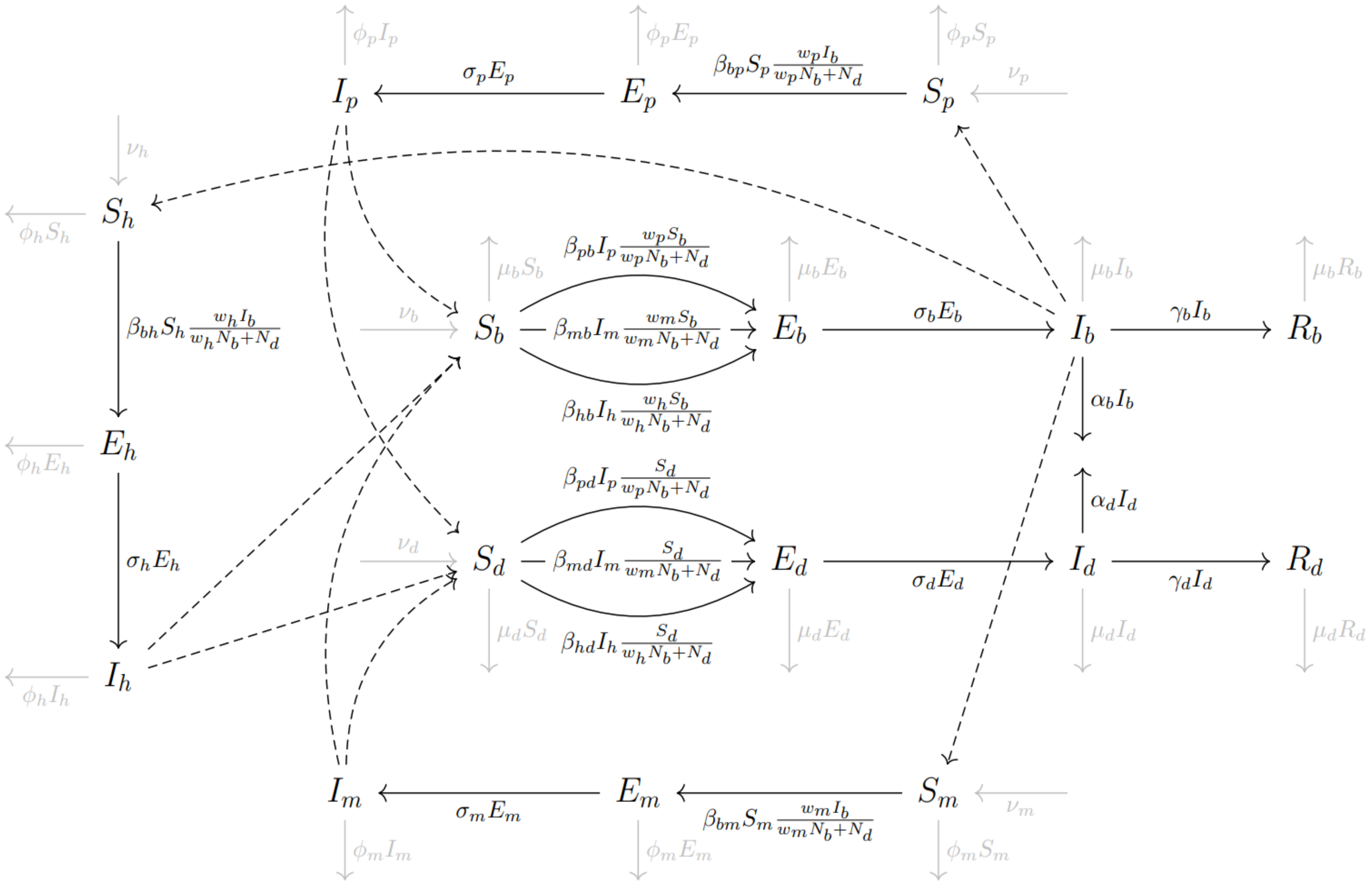

Figure S1. Flow diagram for the multi-vector and multi-host WNV model, with *pipiens* ($N_p = S_p + E_p + I_p$), *molestus* ($N_m = S_m + E_m + I_m$), hybrid ($N_h = S_h + E_h + I_h$), birds as competent ($N_b = S_b + E_b + I_b + R_b$) and humans as dead-end hosts ($N_d = S_d + E_d + I_d + R_d$) contributions. φ_i represents the total mortality acting on mosquitoes, i.e., natural mortality and mortality due to competition (e.g., $\varphi_p = c_{pp}d_{pp}N_p + c_{pm}d_{pm}N_m + c_{ph}d_{ph}N_h + \mu_p$). Black arrows represent compartmental transitions related to infection, grey arrows are demography related movements, and dashed arrows correspond to transmission routes.

The full system of our SEIR model consists of the following differential equations:

$$
\begin{aligned}
N_p' &= \nu_p - (c_{pp}N_p + c_{pm}N_m + c_{ph}N_h + \mu_p)\, N_p \\
N_m' &= \nu_m - \left(c_{mp}N_p + c_{mm}N_m + c_{mh}N_h + \mu_m\right)N_m \\
N_h' &= \nu_h - \left(c_{hp}N_p + c_{hm}N_m + c_{hh}N_h + \mu_h\right)N_h \\
N_b' &= \mu_b - (\mu_b + \alpha_b)N_b \\
N_d' &= \mu_d - (\mu_d + \alpha_d)N_d \\
E_p' &= \frac{\beta_{bp}S_p w_p I_b}{w_pN_b + N_d} - (c_{pp}d_{pp}N_p + c_{pm}d_{pm}N_m + c_{ph}d_{ph}N_h + \mu_p + \sigma_p)E_p \\
E_m' &= \frac{\beta_{bm}S_m w_m I_b}{w_mN_b + N_d} - (c_{mp}d_{mp}N_p + c_{mm}d_{mm}N_m + c_{mh}d_{mh}N_h + \mu_m + \sigma_m)E_m \\
E_h' &= \frac{\beta_{bh}S_h w_h I_b}{w_hN_b + N_d} - (c_{hp}d_{hp}N_p + c_{hm}d_{hm}N_m + c_{hh}d_{hh}N_h + \mu_h + \sigma_h)E_h \\
E_b' &= \frac{\beta_{pb}I_p w_p S_b}{w_pN_b + N_d} - \frac{\beta_{mb}I_m w_m S_b}{w_mN_b + N_d} - \frac{\beta_{hb}I_h w_h S_b}{w_hN_b + N_d} - (\mu_b + \sigma_b)E_b \\
E_d' &= \frac{\beta_{pd}I_p S_d}{w_pN_b + N_d} - \frac{\beta_{md}I_m S_d}{w_mN_b + N_d} - \frac{\beta_{hd}I_h S_d}{w_hN_b + N_d} - (\mu_d + \sigma_d)E_d \\
I_p' &= \sigma_p E_p - (c_{pp}d_{pp}N_p + c_{pm}d_{pm}N_m + c_{ph}d_{ph}N_h + \mu_p)I_p \\
I_m' &= \sigma_m E_m - (c_{mp}d_{mp}N_p + c_{mm}d_{mm}N_m + c_{mh}d_{mh}N_h + \mu_m)I_m \\
I_h' &= \sigma_h E_h - (c_{hp}d_{hp}N_p + c_{hm}d_{hm}N_m + c_{hh}d_{hh}N_h + \mu_h)I_h \\
I_b' &= \sigma_b E_b - (\mu_b + \alpha_b + \gamma_b)I_b \\
I_d' &= \sigma_d E_d - (\mu_d + \alpha_d + \gamma_d)I_d \\
R_b' &= \gamma_b I_b - \mu_b R_b \\
R_d' &= \gamma_d I_d - \mu_d R_d
\end{aligned}
$$

To calculate R_0, we identify the states-at-infection within the aforementioned system and construct matrices of transmissions ***T***, and transitions ***Σ***, as follows:

$$
\boldsymbol{T} = \begin{pmatrix}
0 & 0 & 0 & 0 & 0 & 0 & 0 & 0 & \frac{\beta_{bp}N_p w_p}{w_pN_b + N_d} & 0 \\
0 & 0 & 0 & 0 & 0 & 0 & 0 & 0 & \frac{\beta_{bm}N_m w_m}{w_mN_b + N_d} & 0 \\
0 & 0 & 0 & 0 & 0 & 0 & 0 & 0 & \frac{\beta_{bh}N_h w_h}{w_hN_b + N_d} & 0 \\
0 & 0 & 0 & 0 & 0 & \frac{\beta_{pb}N_b w_p}{w_pN_b + N_d} & \frac{\beta_{mb}N_b w_m}{w_mN_b + N_d} & \frac{\beta_{hb}N_b w_h}{w_hN_b + N_d} & 0 & 0 \\
0 & 0 & 0 & 0 & 0 & \frac{\beta_{pd}N_d}{w_pN_b + N_d} & \frac{\beta_{md}N_d}{w_mN_b + N_d} & \frac{\beta_{hd}N_d}{w_hN_b + N_d} & 0 & 0 \\
0 & 0 & 0 & 0 & 0 & 0 & 0 & 0 & 0 & 0 \\
0 & 0 & 0 & 0 & 0 & 0 & 0 & 0 & 0 & 0 \\
0 & 0 & 0 & 0 & 0 & 0 & 0 & 0 & 0 & 0 \\
0 & 0 & 0 & 0 & 0 & 0 & 0 & 0 & 0 & 0 \\
0 & 0 & 0 & 0 & 0 & 0 & 0 & 0 & 0 & 0
\end{pmatrix}
$$

And:

$$\boldsymbol{\Sigma} = \begin{pmatrix} \Sigma_{11} & 0 & 0 & 0 & 0 & 0 & 0 & 0 & 0 & 0 \\ 0 & \Sigma_{22} & 0 & 0 & 0 & 0 & 0 & 0 & 0 & 0 \\ 0 & 0 & \Sigma_{33} & 0 & 0 & 0 & 0 & 0 & 0 & 0 \\ 0 & 0 & 0 & -\mu_b - \sigma_b & 0 & 0 & 0 & 0 & 0 & 0 \\ 0 & 0 & 0 & 0 & -\mu_d - \sigma_d & 0 & 0 & 0 & 0 & 0 \\ \sigma_p & 0 & 0 & 0 & 0 & \Sigma_{66} & 0 & 0 & 0 & 0 \\ 0 & \sigma_m & 0 & 0 & 0 & 0 & \Sigma_{77} & 0 & 0 & 0 \\ 0 & 0 & \sigma_h & 0 & 0 & 0 & 0 & \Sigma_{88} & 0 & 0 \\ 0 & 0 & 0 & \sigma_b & 0 & 0 & 0 & 0 & -\mu_b - \alpha_b - \gamma_b & 0 \\ 0 & 0 & 0 & 0 & \sigma_d & 0 & 0 & 0 & 0 & -\mu_d - \alpha_d - \gamma_d \end{pmatrix}$$

Where:

$$\Sigma_{11} = -\mu_p - c_{ph}d_{ph}N_h - c_{pm}d_{pm}N_m - c_{pp}d_{pp}N_p - \sigma_p$$
$$\Sigma_{22} = -\mu_m - c_{mh}d_{mh}N_h - c_{mm}d_{mm}N_m - c_{mp}d_{mp}N_p - \sigma_m$$
$$\Sigma_{33} = -\mu_h - c_{hh}d_{hh}N_h - c_{hm}d_{hm}N_m - c_{hp}d_{hp}N_p - \sigma_h$$
$$\Sigma_{66} = -\mu_p - c_{ph}d_{ph}N_h - c_{pm}d_{pm}N_m - c_{pp}d_{pp}N_p$$
$$\Sigma_{77} = -\mu_m - c_{mh}d_{mh}N_h - c_{mm}d_{mm}N_m - c_{mp}d_{mp}N_p$$
$$\Sigma_{88} = -\mu_h - c_{hh}d_{hh}N_h - c_{hm}d_{hm}N_m - c_{hp}d_{hp}N_p$$

The element at position (*i, i*) in ***Σ*** can be interpreted as the rate at which individuals leave compartment *i*. The (*i*, *j*) entry of ***T*** represents the rate at which infected individuals in compartment *j* generate secondary infections in compartment *i*. Multiplying these two matrices provides the next-generation matrix (of large domain) $\boldsymbol{K_L} = -\boldsymbol{T}\boldsymbol{\Sigma}^{-1}$, which is given by:

$$\boldsymbol{K_L} = \begin{pmatrix} 0 & 0 & 0 & k_{14} & 0 & 0 & 0 & 0 & k_{19} & 0 \\ 0 & 0 & 0 & k_{24} & 0 & 0 & 0 & 0 & k_{29} & 0 \\ 0 & 0 & 0 & k_{34} & 0 & 0 & 0 & 0 & k_{39} & 0 \\ k_{41} & k_{42} & k_{43} & 0 & 0 & k_{46} & k_{47} & k_{48} & 0 & 0 \\ k_{51} & k_{52} & k_{53} & 0 & 0 & k_{56} & k_{57} & k_{58} & 0 & 0 \\ 0 & 0 & 0 & 0 & 0 & 0 & 0 & 0 & 0 & 0 \\ 0 & 0 & 0 & 0 & 0 & 0 & 0 & 0 & 0 & 0 \\ 0 & 0 & 0 & 0 & 0 & 0 & 0 & 0 & 0 & 0 \\ 0 & 0 & 0 & 0 & 0 & 0 & 0 & 0 & 0 & 0 \\ 0 & 0 & 0 & 0 & 0 & 0 & 0 & 0 & 0 & 0 \end{pmatrix}$$

Where:

$$k_{14} = \frac{\beta_{bp}N_p\sigma_b w_p}{(\alpha_b + \gamma_b + \mu_b)(\mu_b + \sigma_b)(N_d + N_b w_p)}$$

$$k_{19} = \frac{\beta_{bp}N_p w_p}{(\alpha_b + \gamma_b + \mu_b)(N_d + N_b w_p)}$$

$$k_{24} = \frac{\beta_{bm}N_m\sigma_b w_m}{(\alpha_b + \gamma_b + \mu_b)(\mu_b + \sigma_b)(N_d + N_b w_m)}$$

$$k_{29} = \frac{\beta_{bm}N_m w_m}{(\alpha_b + \gamma_b + \mu_b)(N_d + N_b w_m)}$$

$$k_{34} = \frac{\beta_{bh}N_h\sigma_b w_h}{(\alpha_b + \gamma_b + \mu_b)(\mu_b + \sigma_b)(N_d + N_b w_h)}$$

$$k_{39} = \frac{\beta_{bh}N_h w_h}{(\alpha_b + \gamma_b + \mu_b)(N_d + N_b w_h)}$$

$$k_{41} = \frac{\beta_{pb}N_b\sigma_p w_p}{(\mu_p + c_{ph}d_{ph}N_h + c_{pm}d_{pm}N_m + c_{pp}d_{pp}N_p)(\mu_p + c_{ph}d_{ph}N_h + c_{pm}d_{pm}N_m + c_{pp}d_{pp}N_p + \sigma_p)(N_d + N_b w_p)}$$

$$k_{42} = \frac{\beta_{mb}N_b\sigma_m w_m}{(\mu_m + c_{mh}d_{mh}N_h + c_{mm}d_{mm}N_m + c_{mp}d_{mp}N_p)(\mu_m + c_{mh}d_{mh}N_h + c_{mm}d_{mm}N_m + c_{mp}d_{mp}N_p + \sigma_m)(N_d + N_b w_m)}$$

$$k_{43} = \frac{\beta_{hb} N_b \sigma_h w_h}{(\mu_h + c_{hh} d_{hh} N_h + c_{hm} d_{hm} N_m + c_{hp} d_{hp} N_p)(\mu_h + c_{hh} d_{hh} N_h + c_{hm} d_{hm} N_m + c_{hp} d_{hp} N_p + \sigma_h)(N_d + N_b w_h)}$$

$$k_{46} = \frac{\beta_{pb} N_b w_p}{(\mu_p + c_{ph} d_{ph} N_h + c_{pm} d_{pm} N_m + c_{pp} d_{pp} N_p)(N_d + N_b w_p)}$$

$$k_{47} = \frac{\beta_{mb} N_b w_m}{(\mu_m + c_{mh} d_{mh} N_h + c_{mm} d_{mm} N_m + c_{mp} d_{mp} N_p)(N_d + N_b w_m)}$$

$$k_{48} = \frac{\beta_{hb} N_b w_h}{(\mu_h + c_{hh} d_{hh} N_h + c_{hm} d_{hm} N_m + c_{hp} d_{hp} N_p)(N_d + N_b w_h)}$$

$$k_{51} = \frac{\beta_{pd} N_d \sigma_p}{(\mu_p + c_{ph} d_{ph} N_h + c_{pm} d_{pm} N_m + c_{pp} d_{pp} N_p)(\mu_p + c_{ph} d_{ph} N_h + c_{pm} d_{pm} N_m + c_{pp} d_{pp} N_p + \sigma_p)(N_d + N_b w_p)}$$

$$k_{52} = \frac{\beta_{md} N_d \sigma_m}{(\mu_m + c_{mh} d_{mh} N_h + c_{mm} d_{mm} N_m + c_{mp} d_{mp} N_p)(\mu_m + c_{mh} d_{mh} N_h + c_{mm} d_{mm} N_m + c_{mp} d_{mp} N_p + \sigma_m)(N_d + N_b w_m)}$$

$$k_{53} = \frac{\beta_{hd} N_d \sigma_h}{(\mu_h + c_{hh} d_{hh} N_h + c_{hm} d_{hm} N_m + c_{hp} d_{hp} N_p)(\mu_h + c_{hh} d_{hh} N_h + c_{hm} d_{hm} N_m + c_{hp} d_{hp} N_p + \sigma_h)(N_d + N_b w_h)}$$

$$k_{56} = \frac{\beta_{pd} N_d}{(\mu_p + c_{ph} d_{ph} N_h + c_{pm} d_{pm} N_m + c_{pp} d_{pp} N_p)(N_d + N_b w_p)}$$

$$k_{57} = \frac{\beta_{md} N_d}{(\mu_m + c_{mh} d_{mh} N_h + c_{mm} d_{mm} N_m + c_{mp} d_{mp} N_p)(N_d + N_b w_m)}$$

$$k_{58} = \frac{\beta_{hd} N_d}{(\mu_h + c_{hh} d_{hh} N_h + c_{hm} d_{hm} N_m + c_{hp} d_{hp} N_p)(N_d + N_b w_h)}$$

Each element k_{ij} corresponds to the expected number of new infections in compartment *I* generated by an individual who initially entered the infected state as a member of compartment *j*. This can be simplified to a 5x5 matrix by considering that the individuals in the latent compartment are infected but not yet infectious, and that humans are dead-end hosts. These individuals do not contribute to the further transmission of the disease. Therefore, we reduce the matrix to include only the infectious states, excluding all non-contributing infected states.

First, the matrix ***T*** contains four rows composed entirely of zeros. To handle this, we define an intermediate matrix ***E***, as described in Diekmann et al.[2] as follows:

$$\boldsymbol{E} = \begin{pmatrix} 1 & 0 & 0 & 0 & 0 \\ 0 & 1 & 0 & 0 & 0 \\ 0 & 0 & 1 & 0 & 0 \\ 0 & 0 & 0 & 1 & 0 \\ 0 & 0 & 0 & 0 & 1 \\ 0 & 0 & 0 & 0 & 0 \\ 0 & 0 & 0 & 0 & 0 \\ 0 & 0 & 0 & 0 & 0 \\ 0 & 0 & 0 & 0 & 0 \\ 0 & 0 & 0 & 0 & 0 \end{pmatrix}$$

Next, we obtain the next-generation matrix ***K*** by calculating ***E' K E***, resulting in:

$$\boldsymbol{K} = \begin{pmatrix} 0 & 0 & 0 & k_{14} & 0 \\ 0 & 0 & 0 & k_{24} & 0 \\ 0 & 0 & 0 & k_{34} & 0 \\ k_{41} & k_{42} & k_{43} & 0 & 0 \\ k_{51} & k_{52} & k_{53} & 0 & 0 \end{pmatrix}$$

The dominant eigenvalue of this matrix, evaluated at the disease-free equilibrium, corresponds to R_0:

$$R_0 = \sqrt{k_{14}\, k_{41} + k_{24} k_{42} + k_{34} k_{43}}$$

We can quantify the contributions of each mosquito (eco)type to WNV invasion by setting the contributions of the remaining (eco)types to zero. This results in the following R_0:

$$R_0^p = \sqrt{\frac{\beta_{bp} N_p \sigma_b w_p}{(\alpha_b + \gamma_b + \mu_b)(\mu_b + \sigma_b)(N_d + N_b w_p)} \frac{\beta_{pb} N_b \sigma_p w_p}{(\mu_p + c_{pp} d_{pp} N_p)(\mu_p + c_{pp} d_{pp} N_p + \sigma_p)(N_d + N_b w_p)}}$$

$$R_0^m = \sqrt{\frac{\beta_{bm} N_m \sigma_b w_m}{(\alpha_b + \gamma_b + \mu_b)(\mu_b + \sigma_b)(N_d + N_b w_m)} \frac{\beta_{mb} N_b \sigma_m w_m}{(\mu_m + c_{mm} d_{mm} N_m)(\mu_m + c_{mm} d_{mm} N_m + \sigma_m)(N_d + N_b w_m)}}$$

$$R_0^h = \sqrt{\frac{\beta_{bh} N_h \sigma_b w_h}{(\alpha_b + \gamma_b + \mu_b)(\mu_b + \sigma_b)(N_d + N_b w_h)} \frac{\beta_{hb} N_b \sigma_h w_h}{(\mu_h + c_{hh} d_{hh} N_h)(\mu_h + c_{hh} d_{hh} N_h + \sigma_h)(N_d + N_b w_h)}}$$

corresponding to the contribution of *pipiens*, *molestus*, and hybrids to the basic reproduction number. In other words, R_0^p represents the expected number of secondary infections when *molestus* and hybrids are absent, R_0^m represents the expected number of secondary infections when *pipiens* and hybrids are absent, and R_0^h represents the expected number of secondary infections when *pipiens* and *molestus* are absent.

To provide a comprehensive view, we also calculate the contributions of each pairwise combination of (eco)types to the R_0 as follows:

$$R_0^{pm} = \left[\frac{\beta_{bp} N_p \sigma_b w_p}{(\alpha_b + \gamma_b + \mu_b)(\mu_b + \sigma_b)(N_d + N_b w_p)} \frac{\beta_{pb} N_b \sigma_p w_p}{(\mu_p + c_{pp} d_{pp} N_p + c_{pm} d_{pm} N_m)(\mu_p + c_{pp} d_{pp} N_p + c_{pm} d_{pm} N_m + \sigma_p)(N_d + N_b w_p)} \frac{\beta_{bm} N_m \sigma_b w_m}{(\alpha_b + \gamma_b + \mu_b)(\mu_b + \sigma_b)(N_d + N_b w_m)} \frac{\beta_{mb} N_b \sigma_m w_m}{(\mu_m + c_{mm} d_{mm} N_m)(\mu_m + c_{mm} d_{mm} N_m + \sigma_m)(N_d + N_b w_m)}\right]^{\frac{1}{2}}$$

$$R_0^{mh} = \left[\frac{\beta_{bp} N_p \sigma_b w_p}{(\alpha_b + \gamma_b + \mu_b)(\mu_b + \sigma_b)(N_d + N_b w_p)} \frac{\beta_{pb} N_b \sigma_p w_p}{(\mu_p + c_{pp} d_{pp} N_p + c_{pm} d_{pm} N_m)(\mu_p + c_{pp} d_{pp} N_p + c_{pm} d_{pm} N_m + \sigma_p)(N_d + N_b w_p)} \frac{\beta_{bm} N_m \sigma_b w_m}{(\alpha_b + \gamma_b + \mu_b)(\mu_b + \sigma_b)(N_d + N_b w_m)} \frac{\beta_{mb} N_b \sigma_m w_m}{(\mu_m + c_{mm} d_{mm} N_m)(\mu_m + c_{mm} d_{mm} N_m + \sigma_m)(N_d + N_b w_m)}\right]^{\frac{1}{2}}$$

$$R_0^{ph} = \left[\frac{\beta_{bp} N_p \sigma_b w_p}{(\alpha_b + \gamma_b + \mu_b)(\mu_b + \sigma_b)(N_d + N_b w_p)} \frac{\beta_{pb} N_b \sigma_p w_p}{(\mu_p + c_{pp} d_{pp} N_p + c_{pm} d_{pm} N_m)(\mu_p + c_{pp} d_{pp} N_p + c_{pm} d_{pm} N_m + \sigma_p)(N_d + N_b w_p)} \frac{\beta_{bm} N_m \sigma_b w_m}{(\alpha_b + \gamma_b + \mu_b)(\mu_b + \sigma_b)(N_d + N_b w_m)} \frac{\beta_{mb} N_b \sigma_m w_m}{(\mu_m + c_{mm} d_{mm} N_m)(\mu_m + c_{mm} d_{mm} N_m + \sigma_m)(N_d + N_b w_m)}\right]^{\frac{1}{2}}$$

C) Model parametrization: incorporating vector and host data for WNV transmission analysis

The current study includes data collected in 2013 from 45 distinct sites. We considered three different habitat types, as previously described in Martínez-de la Puente et al.[5]. These habitats exhibit variations in vector and host abundances, as well as differences in the feeding preference rates of vectors. Urban habitats are characterized by higher abundances of *molestus* mosquitoes and a denser human population. Rural habitats displayed a greater

abundance of hybrid mosquitoes compared to *pipiens* or *molestus*, while natural habitats exhibit higher abundances of both *pipiens* mosquitoes and birds, alongside a lower human density, compared to the other two habitat types. It is important to note that our data, collected in southern Spain, may represent an idealized scenario, and the validity of these findings may depend on the specific environmental conditions characteristic of the studied areas.

All the parameter values used for model calibration, including data for vector[5] and host species[6], are presented in Table 2 within the manuscript. Numerical values parametrized were obtained in the framework of different studies. Specifically, data on the feeding preference of *Cx. pipiens* (eco)types were extracted from Martínez-de la Puente et al.[5], while information on the total number of mosquito captures and human densities came from Ferraguti et al.[7]. Briefly, mosquitoes were captured from April to December using BG-sentinel traps baited with dry ice as a source of CO_2 at 45 different sampling sites in Cadiz, Huelva and Seville provinces. The three mosquito types were identified following Bahnck and Fonseca protocol[8], and the vertebrate origin of engorged females was determined using a nested PCR[9].

Human abundance was defined as the number of people within a discontinuous grid measuring 250 x 250 meters. This grid was developed by the Institute of Statistics and Cartography of Andalusia, as described in Ferraguti et al.[7]. Population data for the grid cells were assigned based on the number of residents registered as of January 1st, 2013 (the same year as the mosquito and bird data) in *the Base de Datos Longitudinal de Población de Andalucía*.

Vertebrate censuses, which included birds and other mammals, were conducted bimonthly from April to December 2013 in the areas surrounding the mosquito traps. Birds including house sparrows and all other avian species present, were recorded by using point counts and direct observation methods (visual and bird calls or songs identification). Mammals (excluding humans) were recorded through direct observation and pellet counts. This was carried out along transects measuring 200 meters in length and 1 meter in width in the surroundings of the mosquito traps[6].

---

[5] J. Martínez-de la Puente, M. Ferraguti, S. Ruiz, D. Roiz, R. Soriguer, J. Figuerola, *Culex pipiens* forms and urbanization: Effects on blood feeding sources and transmission of avian *Plasmodium*. Malar J 15 (2016) 589.

[6] M. Ferraguti, J. Martínez-de la Puente, S. Bensch, D. Roiz, S. Ruiz, D.S. Viana, R.C. Soriguer, J. Figuerola, Ecological determinants of avian malaria infections: An integrative analysis at landscape, mosquito and vertebrate community levels. J Anim Eco 87 (2018) 727–740.

[7] M. Ferraguti, J. Martínez-De La Puente, D. Roiz, S. Ruiz, R. Soriguer, J. Figuerola, Effects of landscape anthropization on mosquito community composition and abundance, Sci Rep 6 (2016) 29002.

[8] C. Bahnck, D. Fonseca, Rapid assay to identify the two genetic forms of *Culex (Culex) pipiens* L. (Diptera: Culicidae) and hybrid populations. Am J Trop Med Hyg 75 (2006), 251–255.

[9] M. Alcaide, C. Rico, S. Ruiz, R. Soriguer, J. Muñoz, J. Figuerola Disentangling vector-borne transmission networks: a universal DNA barcoding method to identify vertebrate hosts from arthropod bloodmeals. PLoS One 4 (2009), e7092.

The transmission rates (β_{ij}) were parameterized based on Vogel et al.[10], with each rate representing the average value across the three temperature levels reported in that study. Due to logistical constraints and the considerable costs associated with conducting an exhaustive genetic study to determine the precise abundance of *pipiens, molestus* and hybrid mosquitoes captured, obtaining exact measurements for the abundance of each vector type in every habitat was not feasible. Consequently, the vector abundance was assumed followed the proportions observed in Martínez-de la Puente et al. [5] (Table S3). In all scenarios, we assessed the risk of WNV invasion into the population based on the calculated R_0 value.

[10] C.B.F. Vogels, N. Hartemink, C.J.M. Koenraadt, Modelling West Nile virus transmission risk in Europe: Effect of temperature and mosquito biotypes on the basic reproduction number. Sci Rep 7 (2017) 5022.

SUPPLEMENTARY TABLES

Table S1. Assumed vector abundance per season and habitat. Due to logistical and cost constraints, we estimated vector abundance by adopting proportions from in Martínez-de la Puente et al. [5] rather than conducting a precise genetic study to determine the number of the *Cx. pipiens* (eco)types present in each habitat, as observed in Ferraguti et al. [7].

Season	Variable	Natural	Rural	Urban
Spring*	*Cx. pipiens* (eco)type	957	657	310
	Cx. molestus (eco)type	477	548	1,044
	Cx. hybrids	539	767	620
Summer*	*Cx. pipiens* (eco)type	607	417	196
	Cx. molestus (eco)type	303	348	662
	Cx. hybrids	342	487	393

*Spring: vector abundances estimated for May 3rd to June 7th.
*Summer: vector abundances estimated for July 3rd to August 7th.

Table S2. Mosquito feeding preference indexes (w_i) for the *Cx. pipiens* (eco)types based on data from Martínez-de la Puente et al.[5] and Ferraguti et al.[7]. Assumed values marked with *.

Parameter	Variable description	Natural	Rural	Urban
$\hat{w}_p$	*pipiens* preference on *Passer*	0.01*	0.52	0.96
$\hat{w}_m$	*molestus* preference on *Passer*	0.01*	0.21	0.66
$\hat{w}_h$	Hybrids preference on *Passer*	0.01*	0.75	0.64

Table S3. Vector and host species abundances (N_i), blood-fed mosquitoes of the *Cx. pipiens* (eco)types (c_i), and their bites on house sparrows (n_i), per habitat.

Variable	Description	Natural	Rural	Urban	Reference
N_p	*pipiens* estimated abundance	48.5% (5,138)	33.3% (1,629)	15.7% (594)	[7]
N_m	*molestus* estimated abundance	24.2% (2,569)	27.8% (1,357)	52.9% (2,003)	[7]
N_h	Hybrids estimated abundance	27.3% (2,890)	38.9% (1,900)	31.4% (1,187)	[7]
M_i	Total *Culex* complex mosquitoes captures	100% (10,597)	100% (4,886)	100% (3,784)	[7]
N_b	*Passer domesticus* contacts	2,556	7,075	7,396	[7]
N_d	Human abundance by census	82	176	2,811	[7]
N_t	Total host contacts (all birds and mammals)	18,500	14,829	18,834	[6]
c_p	Total *pipiens* (eco)type blood-fed	16	12	8	[5]
c_m	Total *molestus* (eco)type blood-fed	8	10	27	[5]
c_h	Total hybrid blood-fed mosquitoes	9	14	16	[5]
n_{pb}	*pipiens* (eco)type bites on *Passer domesticus*	0	3	3	[5]
n_{mb}	*molestus* (eco)type bites on *Passer domesticus*	0	1	7	[5]
n_{hb}	hybrid mosquitoes bites on *Passer domesticus*	0	5	4	[5]

Note: The abundance of *pipiens* in the natural habitat was calculated as follows: $N_p = c_p / (c_p + c_m + c_h) * M_n = 0.485 * 10{,}597 = 5{,}138$, where c_i represents the mosquito count of each *pipiens* (eco)type at the natural habitat, and M_n is the total number of captured mosquitoes at the natural habitat. The same estimation method was applied to determine the abundance of the other vector types in different habitats.
